# The use of internet analytics by a Canadian provincial chiropractic regulator to monitor, evaluate and remediate misleading claims regarding specific health conditions, pregnancy, and COVID-19

**DOI:** 10.1186/s12998-020-00314-9

**Published:** 2020-05-11

**Authors:** Greg Kawchuk, Jan Hartvigsen, Stan Innes, J. Keith Simpson, Brian Gushaty

**Affiliations:** 1 University of Alberta, Edmonton, Canada; 2 University of Southern Denmark, Odense, Denmark; 3 Nordic Institute of Chiropractic and Clinical Biomechanics, Odense, Denmark; 4 Murdoch University, Perth, Australia; 5 No Affiliation, Edmonton, Canada

**Keywords:** Chiropractic, Internet analytics, Regulation, Misinformation

## Abstract

**Background:**

Internet analytics are increasingly being integrated into public health regulation. One specific application is to monitor compliance of website and social media activity with respect to jurisdictional regulations. These data may then identify breaches of compliance and inform disciplinary actions. Our study aimed to evaluate the novel use of internet analytics by a Canadian chiropractic regulator to determine their registrants compliance with three regulations related to specific health conditions, pregnancy conditions and most recently, claims of improved immunity during the COVID-19 crisis.

**Methods:**

A customized internet search tool (Market Review Tool, MRT) was used by the College of Chiropractors of British Columbia (CCBC), Canada to audit registrants websites and social media activity. The audits extracted words whose use within specific contexts is not permitted under CCBC guidelines. The MRT was first used in October of 2018 to identify words related to specific health conditions. The MRT was again used in December 2019 for words related to pregnancy and most recently in March 2020 for words related to COVID-19. In these three MRT applications, potential cases of word misuse were evaluated by the regulator who then notified the practitioner to comply with existing regulations by a specific date. The MRT was then used on that date to determine compliance. Those found to be non-compliant were referred to the regulator’s inquiry committee. We mapped this process and reported the outcomes with permission of the regulator.

**Results:**

In September 2018, 250 inappropriate mentions of specific health conditions were detected from approximately 1250 registrants with 2 failing to comply. The second scan for pregnancy related terms of approximately1350 practitioners revealed 83 inappropriate mentions. Following notification, all 83 cases were compliant within the specified timeframe. Regarding COVID-19 related words, 97 inappropriate mentions of the word “immune” were detected from 1350 registrants with 7 cases of non-compliance.

**Conclusion:**

Internet analytics are an effective way for regulators to monitor internet activity to protect the public from misleading statements. The processes described were effective at bringing about rapid practitioner compliance. Given the increasing volume of internet activity by healthcare professionals, internet analytics are an important addition for health care regulators to protect the public they serve.

## Background

On March 11, 2020, the World Health Organization (WHO) declared the COVID-19 outbreak to be a pandemic [1]. Appropriately, the global health care community responded by emphasising how to combat the disease through frequent hand-washing, sanitizing, and social distancing [2]. At the present time, WHO has stated that no vaccine has been identified for COVID-19 and there are no known interventions effective in preventing, or treating, a COVID-19 infection.

Unfortunately, some clinicians have used the COVID-19 pandemic to promote misinformation whether intentionally or otherwise. For example, cases have been identified where chiropractors have used their internet presence to promote interventions they claim will boost immunity [3, 4]. Such claims are wholly unsubstantiated as outlined in a recent report from the World Federation of Chiropractic [5]; a point emphasized emphatically by multiple national associations globally [6–14]. Without a doubt, the chiropractic profession recognizes this misinformation as potentially dangerous to the public and damaging to professional credibility [15–17].

In most countries, health care professions like chiropractic are overseen by a regulatory body whose duties include registration of members, ensuring ongoing competency and public protection. In protecting the public in British Columbia, Canada, the registrar investigates, adjudicates and forwards any noncompliant activities to its inquiry committee.

In identifying unsubstantiated claims and statements promoted by its registrants, regulatory bodies have traditionally relied on public surveillance to monitor and report potential missteps. Whereas this traditional approach may have dealt with a relatively low volume of regulatory incursions in the past (e.g. evaluation of printed media only), the current volume at which a profession can market itself electronically requires a parallel improvement in regulation. This is especially the case if the volume of complaints remains low and therefore less easy for the public to identify. Thus today, a passive regulatory approach of relying solely on public input may be insufficient given the rapidity that information is spread on the internet.

Despite electronic communications providing new challenges for regulators, this same technology also provides a potential solution. One such solution is technology that searches specific websites and social media accounts. This technology can be modified to proactively monitor activity then allow regulatory bodies to construct timely interventions [18, 19]. While this approach is still evolving in many sectors, proactive monitoring has been used effectively within law enforcement to monitor and detect illegal activity on the internet for decades and continues to be recommended [20–22]. A similar approach for monitoring healthcare professions could address many of the issues that arise with traditional, time-consuming methods of regulation that rely solely on public reporting [23].

Recently, the College of Chiropractors of British Columbia (CCBC) became the first chiropractic regulatory body that we are aware of to use internet analytics in their regulation of registrants. Specifically, the CCBC commissioned software to identify internet content of their registrants that may contravene their established regulations to facilitate rapid remediation of unacceptable advertising.

The CCBC’s use of internet analytics also provides researchers with a unique opportunity to better describe the frequency and content of registrant internet activity and to evaluate the effectiveness of the regulatory process in remediating these claims – something not yet reported in the literature. Should active use of internet analytics be effective in professional regulation, other jurisdictions may consider adopting this approach to enhance public safety.

In this paper, we report on internet analytics by the CCBC to monitor, evaluate and remediate potentially misleading health claims at three discrete time periods: when internet analytics were first employed by the CCBC in 2018 to identify inappropriate claims related to specific health conditions, then again in 2019 for specific claims related to pregnancy care and most recently in 2020 to identify inappropriate claims during the COVID-19 pandemic (details below).

## Methods

### MRT processes

Customized software was commissioned by the CCBC to provide internet analytics of their registrants and was delivered to the College in 2018 (Compliance Verification Tools, Vancouver, British Columbia, Canada). This software, known as the Marketing Review Tool (MRT), scans websites and social media activity of all chiropractors in British Columbia. The software specifically searches for pre-defined words from a target list approved and maintained by the CCBC. Using this target list, searches performed by the MRT are made twice per month for websites, daily for social media and on demand as required. Following a search, the software returns cases where a word from the target list is identified. The CCBC then manually categorizes these cases into A) acceptable use of the target word (e.g. “our office is closed during the COVID crisis”) and B) unacceptable use (e.g. "chiropractic can boost immunity). For any case deemed unacceptable, the chiropractor(s) registered with the website or social media account is sent a notice to remediate the questionable material immediately (e.g. deletion of content). A MRT scan can then be performed multiple times to determine the rate at which compliance is achieved. When the stated deadline for mandatory compliance is reached, a follow up scan can then be performed and final compliance determined. Any non-compliant activity is then dealt with by the CCBC who forwards these cases to its inquiry committee.

### MRT use with specific conditions (October 2018)

The MRT was first used by the CCBC to review the internet activity of their registrants in relation to specific health conditions not permitted to be promoted or treated by registrants. These conditions were made known to registrants on October 3, 2018 with mandatory compliance to occur by November 1, 2018. The regulation providing the targeted words states:

*As stated in section 14(1)(f) of the CCBC’s Professional Conduct Handbook (“PCH”), chiropractors must not advertise health benefits of their services when there is no acceptable evidence that those benefits can be achieved. See Appendix “N” to the Handbook and the Efficacy Claims Policy for additional information.*

*The Board is concerned registrants may be making claims in marketing or directly to patients that chiropractic care has beneficial effects on some diseases, disorders and conditions when there is no acceptable evidence for those claims. This policy identifies efficacy claims that are not supported by acceptable evidence, and therefore, must not be made.* *https://www.chirobc.com/efficacy-claims-policy/*

*Due to the absence of acceptable evidence supporting such claims, registrants must NOT represent to patients or the public that chiropractic: (a) can be used to treat diseases, disorders or conditions such as: Alzheimer’s disease, cancer, diabetes, infections, infertility, or Tourette’s syndrome, or (b) has any beneficial effect on childhood diseases, disorders or conditions such as: ADHD (or ADD), autism spectrum disorders including Asperger syndrome, cerebral palsy, Down syndrome, fetal alcohol syndrome, or developmental and speech disorders.*

A scan of registrant internet analytics was then performed by the MRT in October, before the compliance deadline. Registrants associated with inappropriate messaging were notified before the compliance deadline so they could take corrective action. A subsequent scan was then performed on the compliance date and noncompliant cases were notified and forwarded to the CCBC inquiry committee.

### MRT use with pregnancy conditions (December 2019)

On December 23, 2019, the CCBC released amendments to the Professional Conduct Handbook and the Efficacy Claims Policy regarding pregnancy. The regulation can be found here (https://www.chirobc.com/amendments-to-the-professional-conduct-handbook-and-efficacy-claims-policy-webster-technique-and-pregnancy-related-conditions/) and states:

*Due to the absence of acceptable evidence supporting such claims, registrants must NOT represent to patients or the public that chiropractic: (a) has any beneficial effect on fetal development or position such as: breech/breech turning or position and intrauterine/*in utero *constraint. (b) has any beneficial effect on labour or birth such as: easier or shorter labour, preventing the need for medical interventions and preventing premature or traumatic birth. (c) has any beneficial effect on hormone function or postpartum depression.*

After the adoption of this policy and subsequent notification of registrants, internet analytics based on selected pregnancy target words were generated from the MRT on December 23, 2019 prior to the compliance deadline of January 30, 2020. Registrants associated with inappropriate messaging were notified before the compliance deadline so they could take corrective action. A subsequent scan was then performed on the compliance date and noncompliant cases were notified and forwarded to the CCBC inquiry committee.

### MRT use with COVID-19 (march 2020)

On March 13, 2020, target words were added to the MRT system in response to the COVID pandemic with the expected date of compliance set to the same day. Adding these words to the target list did not require new regulations be passed by the CCBC as claims related to infectious disease were already disallowed through existing regulations.

*The PCH states: 9.5 The prevention and treatment of infectious disease is not within the scope of chiropractic practice.*

The CCBC also released an announcement to the public that claims promoting treatment or supplements to improve immunity were inappropriate. (https://www.chirobc.com/novel-coronavirus-covid-19/) A surveillance scan was performed on March 18, 2020 for words such as COVID, corona, and immune and derivative words. Noncompliant cases were notified to take immediate corrective action. Further scans were then performed and noncompliant cases were notified and forwarded to the CCBC inquiry committee by March 31, 2020.

### Data acquisition

In all three applications of the MRT, our research team was provided with anonymized, aggregated data from the CCBC beginning March 24, 2020 and ending March 31, 2020. This anonymized data (provided by the CCBC with permission given for analysis) consisted solely of numerical totals and dates for all three MRT applications: estimates of the number of websites/social media accounts reviewed by MRT, subsequent cases of potentially inappropriate or non-compliant word use, estimates of the number of registrants and the number of non-compliant cases. Approval for this project was provided by the University of Alberta Human Research Ethics Board (Pro00099878).

## Results

### Specific health conditions

**Fig. 1:**
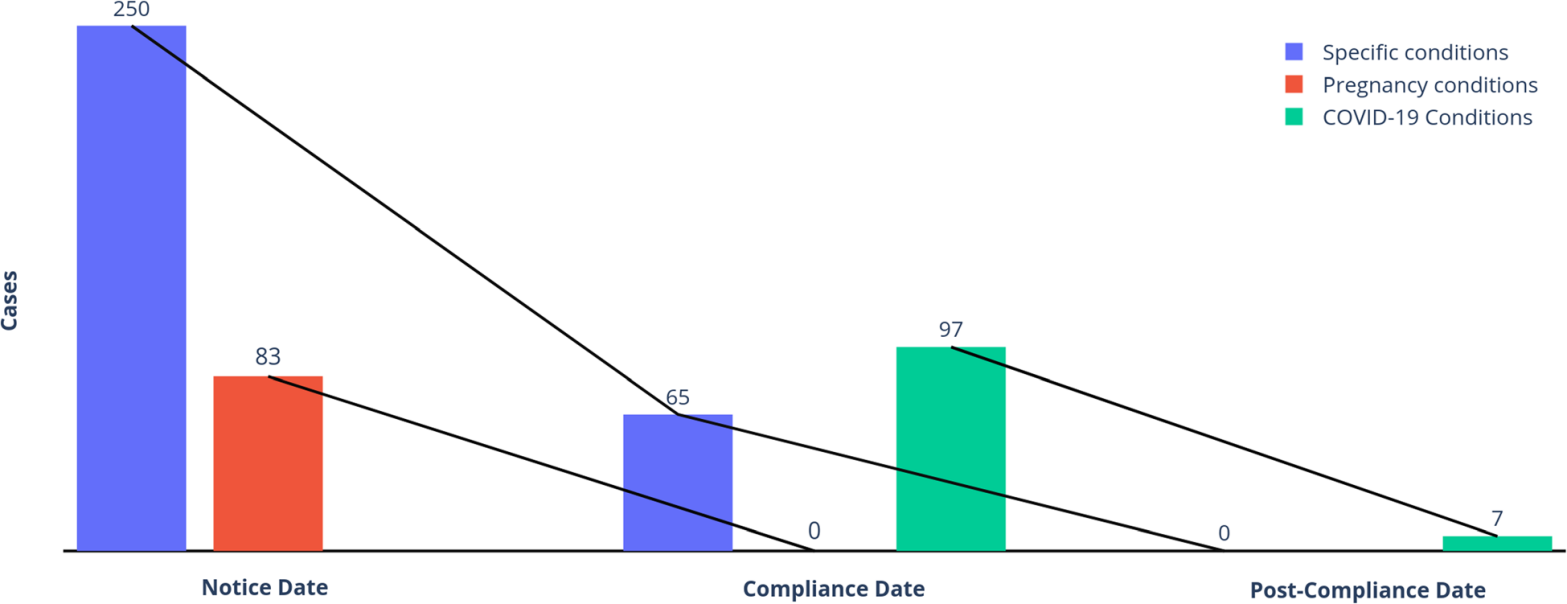
Number of cases of inappropriate mentions over time for three specific applications of software used to provide internet analytics of CCBC registrants

Approximately 750 websites and 650 social media pages of approximately 1250 CCBC registrants were reviewed with the MRT resulting in 250 potentially inappropriate communications (Table 1, Fig. 1). A scan was then conducted on the compliance deadline that identified 65 registrants as non-compliant. Subsequent scans were then performed which showed that all non-compliant cases were resolved except for 2 that remained outstanding and were later resolved through the inquiry committee.

**Table 1:** Number of cases of inappropriate mentions over time for three specific applications of MRT used to provide internet analytics of CCBC registrants

	Notice Date	Compliance Date Outstanding	Post Compliance Outstanding
**Specific Conditions**	October 3, 2018	November 1, 2020	December 2019
	250 inappropriate mentions / approximately 1250 registrants	65 inappropriate mentions / approximately 1250 registrants	2 inappropriate mentions / approximately 1250 registrants
**Pregnancy Conditions**	December 23, 2019	January 30, 2020	Unnecessary
	83 inappropriate mentions / approximately 1350 registrants	0 inappropriate mentions / approximately 1350 registrants	
**Immune Conditions**	March 13, 2020	March 13, 2020	March 2020
		97 inappropriate mentions / approximately 1350 registrants	7 inappropriate mentions / approximately 1350 registrants

### Pregnancy related conditions

This MRT scan identified 636 potential cases from approximated 1350 registrants (Table 1, Fig. 1). Further evaluation by the CCBC classified 83 cases as inappropriate. Internet analytics generated by MRT on the compliance date showed full compliance by all registrants.

### COVID-19 and immunity related issues

The MRT scan returned over 4773 potential cases from approximately 1350 registrants. Specifically, there were 1479 potential cases for “corona” and/or “covid” and 2387 cases for “immune” (Table 1, Fig. 1). After review by the CCBC to eliminate acceptable use of target words (e.g. wash your hands frequently during the COVID crisis), 97 cases were inappropriate in their use of the word “immune”. No inappropriate cases were identified for use of “corona” or “covid”. Notices to remove inappropriate material were emailed in March 2020. Subsequent MRT scans showed that the majority of registrants responded promptly and removed questionable content. As of March 31, 2020, there were 7 sites yet to comply and the names of registrants associated with these sites were passed to the CCBC inquiry committee for further investigation.

## Discussion

We describe the novel use of internet analytics to monitor the social media activity of the chiropractic profession in British Columbia, Canada. Data presented demonstrate this technology can effectively monitor vast amounts of internet activity by registrants over a short period enabling the regulatory body to effectively bring about compliance. Although other Canadian professions are now in possession of this software, we are not aware of any prior report in the literature that describes the use of internet analytics to improve health care regulation in the chiropractic profession.

Traditionally, regulators have relied on public-reporting of inappropriate communications to provide input into their regulatory processes. Although we did not receive data regarding the number of public complaints made to the CCBC during the time in which MRT has been in use, the CCBC communicated that there are occasions when a public concern is brought to their attention before the next scheduled MRT scan. These concerns are acted on immediately. Still, it is doubtful that this level of public surveillance can identify the number of potential cases of inappropriate word use compared to MRT scanning. While public input remains important to the regulatory process, a modern regulatory body whose registrants are increasingly engaged with the public on the internet should not rely exclusively on public input to monitor their registrants when internet analytics are now available.

While both the public and internet surveillance components of a modern regulatory approach are important, neither can be effective without significant human effort. On the public side, effort is needed to not only identify and interpret incursions, but also to ensure they are reported to the regulator promptly. The public must be made aware by the regulator of what is appropriate and inappropriate to make safe health care decisions. Similarly, use of internet analytics by a regulator requires commitment to performing and analyzing scans regularly. Either way this information is obtained, the regulator must use its existing processes to the fullest to address potential concerns. This is important for protecting the public and to act as a deterrent for future incursions given a rapidly evolving health care environment where competition for patients is becoming substantially more intense. This is especially true for the chiropractic profession whose integration into mainstream healthcare is evolving but whose record of advertising practices in some jurisdictions is unsatisfactory [24, 25].

There would appear to be no substantial downside for regulators to adopt internet analytics in their daily operations to evaluate registrant compliance of regulatory directives [26] although challenges may exist regarding cost, training, and/or time to process identified cases.

### Limitations

Current scanning techniques used with the MRT software do not detect target words placed over images (e.g. memes) or text/voice content within video sources. Technologies to address these situations are being tested. The MRT software cannot presently scan all Facebook content as only recent posts are available to the scanner; older posts require user scrolling to be visible.

The MRT software is only effective as the target words used in its searches. The software cannot scan for implication or meaning. As the software allows the user to input new target words, the scan can be expanded as needed to include terms for existing or new regulations.

While we have been informed verbally that MRT software has been ordered by health care professions other than chiropractic, we are not aware that the MRT software is being actively employed in these jurisdictions. Widespread use of MRT software and communication between regulatory bodies as they gain experience in internet analytics may further improve its efficiency.

Importantly, MRT scanning also identifies cases from non-chiropractors who are associated with the registrant through a common clinic website or social media account (e.g. physiotherapy, massage, etc). Cases from associated professions are not pursued by the CCBC nor are they passed forward to other regulatory agencies.

Finally, we cannot assume that all chiropractors knowingly post misinformation on the internet. Although not presented here, the authors know of paid subscription services that provide chiropractors and their websites/social media with “news feeds” that may provide questionable content. Still, individual practitioners remain responsible for what is displayed in their name. This places the onus on these services to review their standards of business and become vigilant, evidence-based content providers.

## Conclusion

Internet analytics are an effective way to monitor website and social media activity of registrants. Use of internet analytics through MRT software is useful for regulatory bodies to keep pace with the high volume of internet activity produced by their registrants and may bring about rapid compliance to existing regulations.

## Data Availability

All data generated or analysed during this study are included in this published article.

## Abbreviations

CCBC: College of chiropractors of British Columbia
MRT: Marketing Review Tool
